# Low-Grade Appendiceal Mucinous Neoplasm Presenting as an Appendiceal Mucocele: A Case Report

**DOI:** 10.7759/cureus.109700

**Published:** 2026-05-26

**Authors:** Marisela Silva García, Hugo E Mora Moreno, Cristian Sandoval Cervantes, Ulises S Sanchez Guevara

**Affiliations:** 1 General Surgery, Hospital General "Dr. Miguel Silva", Morelia, MEX

**Keywords:** appendiceal mucocele, appendiceal neoplasm, low-grade appendiceal mucinous neoplasm, pseudomyxoma peritonei, right hemicolectomy

## Abstract

Appendiceal mucocele is an uncommon clinical and radiological finding characterized by cystic dilatation of the appendix due to intraluminal mucin accumulation and may be associated with underlying epithelial mucinous neoplasms. We present the case of a 45-year-old man with obesity who presented with a four-month history of intermittent right-lower-quadrant abdominal pain, occasional abdominal distension, and nonspecific changes in bowel habits. Physical examination revealed localized tenderness without signs of peritoneal irritation, and laboratory findings were within normal limits. Abdominal ultrasonography demonstrated a cystic tubular structure in the right iliac fossa. Contrast-enhanced computed tomography of the abdomen and pelvis showed a dilated appendix measuring approximately 22 mm in diameter, with homogeneous low-attenuation intraluminal content, wall thickening, and mural calcification, without radiological evidence of perforation, abscess, ascites, or invasion of adjacent structures. Based on these findings, an appendiceal mucocele without radiological signs of complication was suspected. Because the lesion was closely associated with the cecal region and limited appendectomy was considered unsafe due to the risk of rupture or incomplete gross resection, the patient underwent elective open right hemicolectomy with stapled ileotransverse anastomosis. Careful surgical handling was performed to avoid appendiceal rupture and mucin spillage. The postoperative course was uneventful. Histopathological examination confirmed a low-grade appendiceal mucinous neoplasm (LAMN) measuring 10 cm in length and 2.2 cm in diameter, with an uninvolved appendiceal base and no extra-appendiceal mucin, microscopic perforation, invasive adenocarcinoma, or high-grade malignant features. This case highlights the importance of multidisciplinary assessment and risk-adapted follow-up in patients with suspected appendiceal mucinous lesions.

## Introduction

Appendiceal mucoceles are uncommon lesions characterized by cystic dilatation of the appendix due to intraluminal mucin accumulation. Although the term “mucocele” is descriptive rather than a definitive pathological diagnosis, it may be associated with a spectrum of underlying conditions, ranging from non-neoplastic obstruction to epithelial mucinous neoplasms. Among these, low-grade appendiceal mucinous neoplasm (LAMN) represents a clinically relevant entity because of its distinctive histopathological features and its potential association with extra-appendiceal mucin and pseudomyxoma peritonei when rupture or peritoneal dissemination occurs [1,2].

The clinical presentation of appendiceal mucinous lesions is often nonspecific. Patients may be asymptomatic or present with vague abdominal discomfort, chronic right lower quadrant pain, abdominal distension, or symptoms that mimic acute appendicitis or other ileocecal diseases. For this reason, preoperative diagnosis may be challenging and frequently depends on imaging findings. Ultrasonography may identify a cystic tubular structure in the right lower quadrant, whereas computed tomography is useful for assessing appendiceal dilatation, mural calcification, wall characteristics, periappendiceal inflammation, and signs of perforation or peritoneal disease [1,3].

Histopathological evaluation remains essential for establishing the definitive diagnosis and distinguishing LAMN from other appendiceal epithelial lesions. Complete evaluation of the appendix is particularly important because mucinous, serrated, and diverticular changes may overlap morphologically and may influence diagnostic interpretation [4]. When LAMN is confined to the appendix and no high-risk pathological features are identified, the prognosis is generally favorable; however, accurate pathological assessment is necessary to identify findings, such as perforation, extra-appendiceal mucin, base involvement, positive margins when applicable, high-grade cytology, or invasive adenocarcinoma [5].

Surgical management should be individualized according to intraoperative findings, appendiceal integrity, the relationship of the lesion to the appendiceal base or cecum, the feasibility of complete resection, and the presence or absence of peritoneal dissemination. Although appendectomy may be sufficient for selected localized, non-perforated appendiceal mucinous lesions, more extensive resection may be required when the lesion is closely associated with the cecum or when safe complete resection cannot be achieved without risking rupture or mucin spillage. Contemporary guidelines emphasize careful handling of suspected appendiceal mucinous lesions to avoid rupture and peritoneal dissemination, as well as multidisciplinary decision-making when neoplastic disease is confirmed [3]. Postoperative surveillance should also be risk-adapted, as recent evidence suggests that recurrence after resection of LAMN is overall uncommon but may occur in selected higher-risk patients, supporting the need for individualized follow-up strategies [6]. We present the case of a 45-year-old man with chronic right lower quadrant pain and imaging findings suggestive of appendiceal mucocele, whose final histopathological diagnosis was LAMN after elective open right hemicolectomy.

## Case presentation

A 45-year-old man presented for medical evaluation with a four-month history of intermittent right-lower-quadrant abdominal pain. The pain was mild to moderate in intensity, pressure-like in character, non-radiating, and occasionally worsened after food intake and physical activity. He also reported intermittent abdominal distension and nonspecific changes in bowel habits. He denied gastrointestinal bleeding, fever, nausea, vomiting, and unintentional weight loss.

His medical history was notable only for obesity, with a body mass index of 32 kg/m². He had no relevant prior surgical history or other significant comorbidities.

On physical examination, the patient was hemodynamically stable. The abdomen was soft and non-distended, with tenderness to deep palpation in the right lower quadrant. There was no guarding, rebound tenderness, clinical evidence of peritoneal irritation, or palpable abdominal mass.

Initial laboratory findings were within normal limits, with no evidence of leukocytosis, anemia, thrombocytopenia, liver enzyme abnormalities, hyperglycemia, or elevated inflammatory markers (Table 1). Preoperative tumor markers, including carcinoembryonic antigen, carbohydrate antigen 19-9, and cancer antigen 125, were not obtained; postoperative tumor marker assessment was planned according to the final pathological risk profile.

**Table 1:** Initial laboratory findings on presentation.

Parameter	Result	Reference range
White blood cell count	8.4 × 10³/µL	4.5-10.0 × 10³/µL
Hemoglobin	15.3 g/dL	13.5-17.5 g/dL
Platelet count	287 × 10³/µL	150-400 × 10³/µL
Lactate dehydrogenase	141 U/L	120-300 U/L
C-reactive protein	1.2 mg/L	0-6 mg/L
Aspartate aminotransferase	29 U/L	0-40 U/L
Alanine aminotransferase	32 U/L	0-41 U/L
Glucose	87 mg/dL	74-109 mg/dL

Initial abdominal ultrasonography was reported to show a cystic tubular structure in the right iliac fossa, raising suspicion for an appendiceal lesion; however, the original ultrasonographic images were not available for publication. Contrast-enhanced computed tomography of the abdomen and pelvis subsequently revealed a dilated appendix measuring approximately 22 mm in diameter, with homogeneous low-attenuation intraluminal content, wall thickening, and mural calcification. There was no imaging evidence of perforation, periappendiceal abscess, ascites, peritoneal implants, or invasion of adjacent structures. These CT findings were suggestive of an appendiceal mucocele without radiological signs of complication (Figures 1, 2).

**Figure 1:**
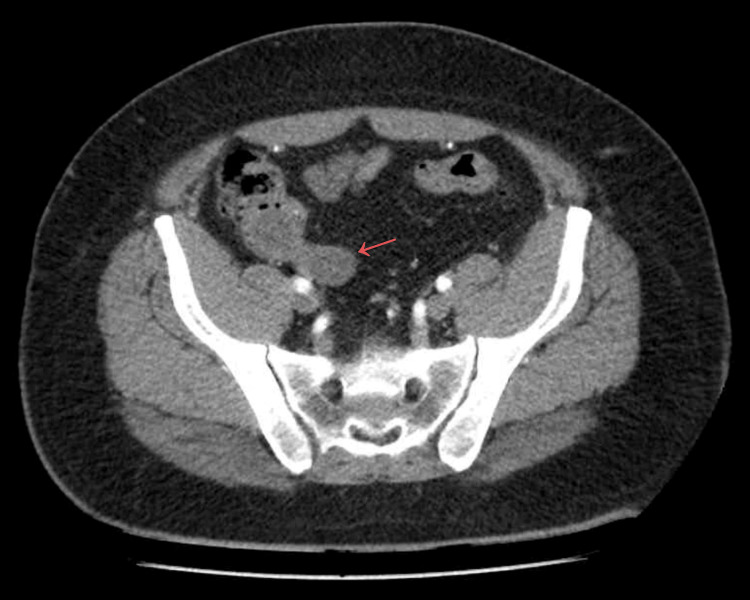
Axial contrast-enhanced computed tomography of the abdomen and pelvis. Axial image showing a cystic tubular structure in the right lower quadrant, corresponding to a dilated appendix with homogeneous low-attenuation intraluminal content. The red arrow indicates the suspected appendiceal mucocele.

**Figure 2:**
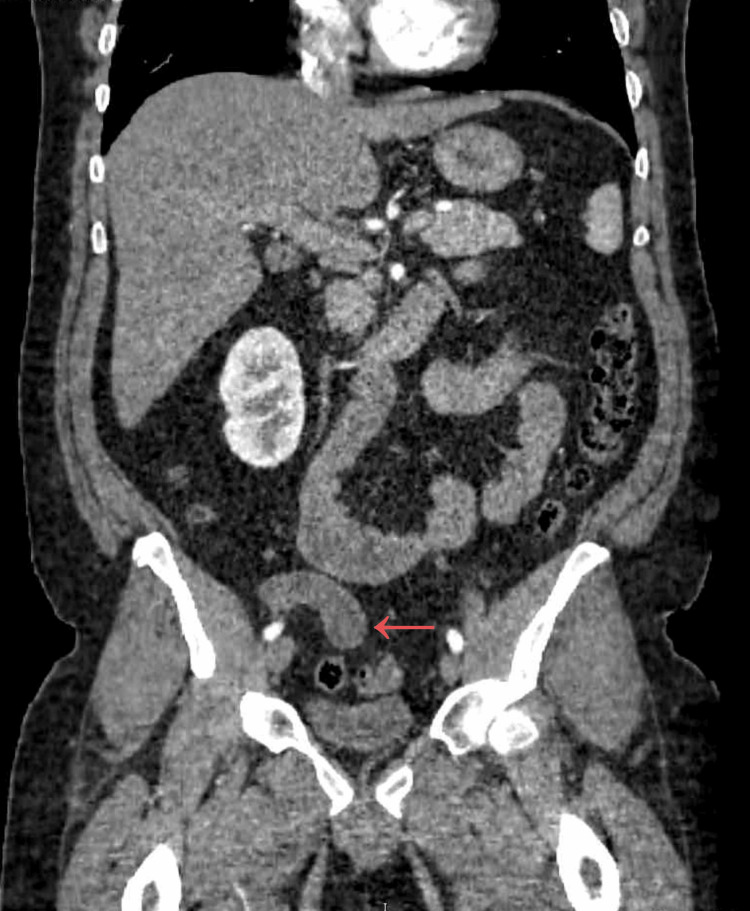
Coronal contrast-enhanced computed tomography of the abdomen and pelvis. Coronal image showing appendiceal dilatation in the right lower quadrant. The red arrow highlights the dilated appendiceal lesion. No radiological signs of perforation, abscess formation, ascites, or invasion of adjacent structures were identified.

Based on the clinical and radiological findings, a working preoperative diagnosis of appendiceal mucocele without radiological signs of complication was made, and the patient was scheduled for elective surgical management.

An open midline laparotomy was performed, and the patient underwent elective open right hemicolectomy with stapled ileotransverse anastomosis. Intraoperatively, a markedly dilated cystic appendix was identified in close relationship with the cecal region, with a gross appearance consistent with an appendiceal mucocele (Figure 3). There was no macroscopic evidence of appendiceal perforation, mucin spillage, peritoneal implants, or disseminated mucinous disease. Because a limited appendectomy was considered unsafe due to the risk of rupture and incomplete gross resection, an open right hemicolectomy with stapled ileotransverse anastomosis was performed to achieve complete resection while minimizing the risk of appendiceal rupture and mucin spillage. The resected surgical specimen included the terminal ileum, right colon, and markedly dilated appendix, supporting complete gross resection of the appendiceal lesion (Figure 4). An abdominal drain was placed, and the procedure was completed without intraoperative complications.

**Figure 3:**
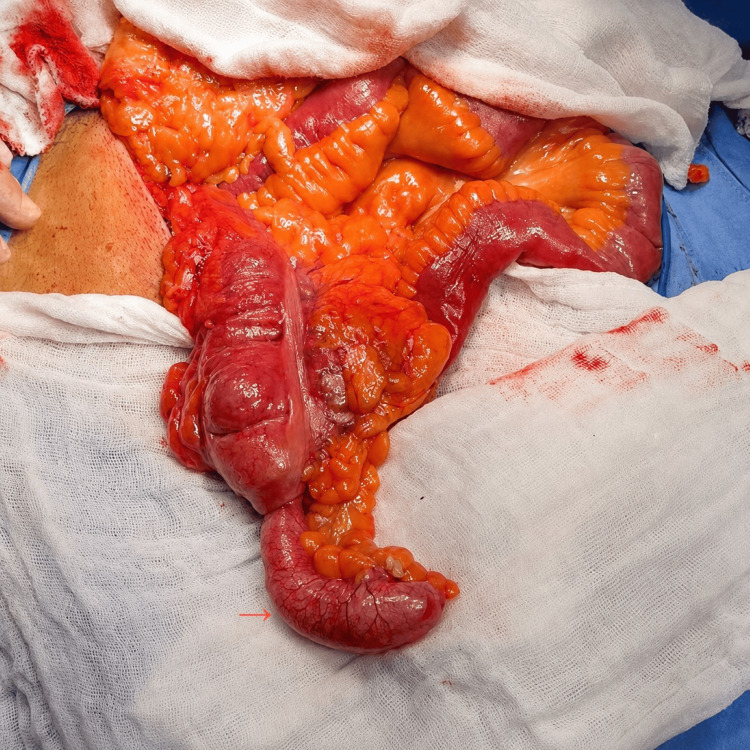
Intraoperative appearance of the dilated appendix. Intraoperative view during open laparotomy showing a markedly dilated cystic appendix in close relationship with the cecal region. The red arrow indicates the appendiceal lesion. No gross rupture or mucin spillage is evident in the visible surgical field.

**Figure 4:**
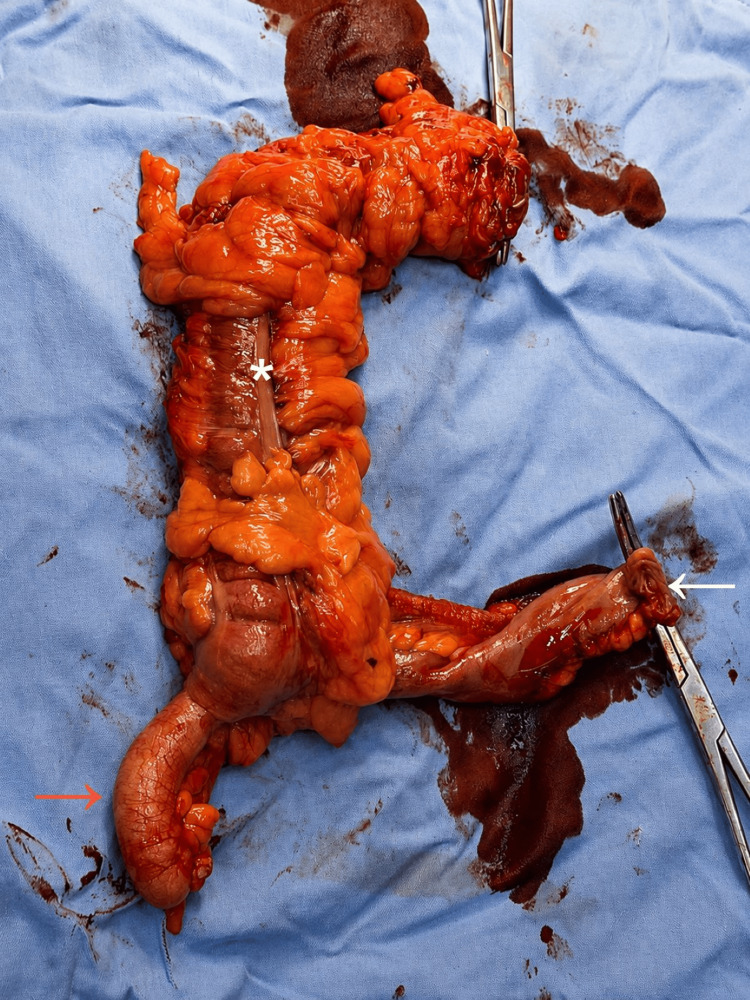
Gross surgical specimen showing the resected appendix. Gross surgical specimen obtained after right hemicolectomy, including the terminal ileum, right colon, and markedly dilated appendix. The red arrow indicates the resected appendix, the white arrow indicates the terminal ileum, and the white asterisk marks the right colon.

The patient remained hospitalized for postoperative monitoring. Oral intake was withheld for 72 hours and was then gradually resumed, with adequate tolerance. Bowel function returned appropriately. The abdominal drain showed serous output, with no evidence of enteric leakage. Given the favorable postoperative course, the patient was discharged home.

Histopathological examination showed replacement of the appendiceal mucosa by flat-to-villous mucinous epithelium, without high-grade cytologic atypia or increased mitotic activity. Representative hematoxylin and eosin-stained imaging demonstrated mucinous epithelial proliferation with villous architecture (Figure 5). Intraluminal mucin was identified. The appendiceal lesion measured 10 cm in length and 2.2 cm in diameter, and the appendiceal base was uninvolved. No extra-appendiceal mucin, microscopic perforation, serosal involvement, lymphovascular invasion, perineural invasion, invasive adenocarcinoma, or high-grade malignant features were identified in the examined specimen. The final pathological diagnosis was LAMN. The available pathological report did not include a regional lymph node assessment.

**Figure 5:**
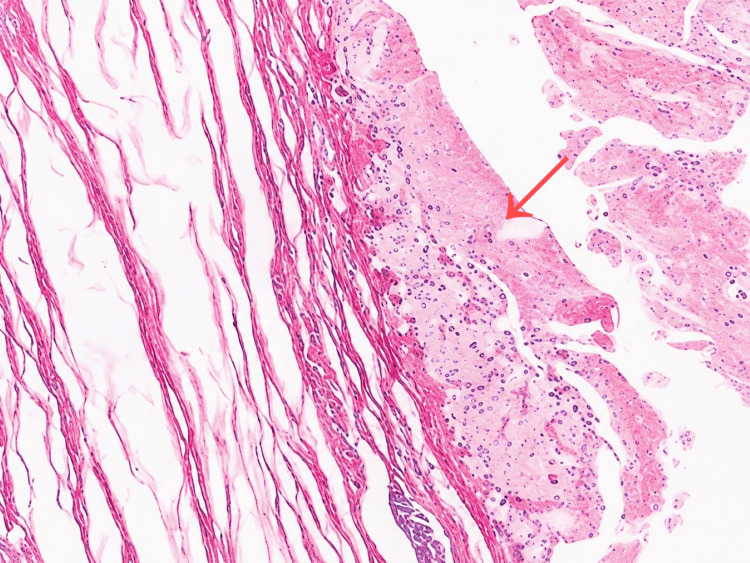
Histopathological findings of low-grade appendiceal mucinous neoplasm. Representative hematoxylin and eosin-stained section of the appendiceal lesion showing mucinous epithelial proliferation with flat-to-villous architecture and low-grade cytologic features. The red arrow indicates the mucinous epithelial proliferation. Final pathological evaluation confirmed low-grade appendiceal mucinous neoplasm, without high-grade malignant features or invasive adenocarcinoma.

At the two-week postoperative follow-up visit, the patient demonstrated adequate clinical recovery. He reported normal bowel movements and denied abdominal pain or other gastrointestinal symptoms. The surgical sutures were removed, and no postoperative complications were identified. Postoperative colonoscopic evaluation was recommended to assess for synchronous colorectal lesions as part of the patient’s follow-up plan. Given the diagnosis of LAMN, the patient was referred for risk-adapted surveillance, including clinical evaluation, tumor marker assessment, and cross-sectional imaging according to institutional protocols and multidisciplinary recommendations.

Written informed consent was obtained from the patient for the publication of this case report and all accompanying clinical, radiological, intraoperative, and surgical specimen images.

## Discussion

Appendiceal neoplasms are uncommon and heterogeneous lesions whose clinical behavior and management depend primarily on histopathological classification and disease extent. This case illustrates an LAMN presenting as an appendiceal mucocele in a 45-year-old man with chronic, nonspecific right-lower-quadrant abdominal pain and normal laboratory findings. Although appendiceal mucoceles may initially be suspected based on imaging, the final diagnosis requires pathological confirmation because the term “mucocele” describes appendiceal dilatation caused by mucin accumulation rather than a single histological entity [7]. Recent surgical series have reported that appendiceal neoplasms represent a small but important proportion of appendectomy specimens, emphasizing the need for careful radiological, intraoperative, and pathological assessment [8]. Clinical presentation may overlap with acute appendicitis, chronic appendiceal inflammation, or other ileocecal conditions; therefore, diagnostic suspicion should be maintained when symptoms are atypical, prolonged, or associated with imaging abnormalities [9]. Systematic evidence also suggests that appendiceal neoplasms may be identified across different clinical presentations, rather than exclusively in patients with severe inflammatory disease [10].

From a diagnostic standpoint, distinguishing a radiological mucocele from an underlying mucinous neoplasm is essential. Appendiceal neoplasms include several pathological entities with different prognostic and therapeutic implications, and LAMN has particular clinical importance because rupture or extra-appendiceal mucin may lead to pseudomyxoma peritonei [11]. Imaging played a central role in the diagnosis and operative planning in this case. Ultrasonography demonstrated a cystic tubular structure in the right iliac fossa, while contrast-enhanced computed tomography showed appendiceal dilatation of approximately 22 mm, homogeneous low-attenuation intraluminal content, wall thickening, and mural calcification. These findings are consistent with imaging features that should raise suspicion for LAMN, particularly when a dilated appendix is identified with mural calcification and without prominent surrounding inflammatory changes [12].

The main surgical objective in suspected appendiceal mucinous lesions is complete resection while avoiding rupture and mucin spillage. This principle is critical because perforation may allow mucin, with or without neoplastic epithelium, to spread into the peritoneal cavity. In the present case, no macroscopic perforation, mucin spillage, peritoneal implants, or disseminated mucinous disease was identified, and the specimen was handled carefully throughout the procedure. This intraoperative strategy is consistent with contemporary case-based evidence emphasizing careful surgical manipulation to reduce the risk of peritoneal dissemination [13]. The extent of surgical resection for LAMN remains a subject of clinical debate and should be individualized according to intraoperative findings, appendiceal integrity, tumor location, relationship to the appendiceal base or cecum, and the feasibility of achieving safe complete resection. Appendectomy may be adequate for selected localized, non-perforated LAMNs; however, more extensive resection may be appropriate when limited appendectomy is considered unsafe or when adequate gross clearance cannot be achieved without risking rupture [14]. In this patient, right hemicolectomy was selected because the dilated appendiceal lesion was closely associated with the cecal region, allowing complete resection of the ileocecal segment while minimizing the risk of appendiceal rupture and mucin spillage [15].

Histopathological examination remains the cornerstone for definitive diagnosis and risk stratification. In this case, the appendiceal mucosa was replaced by flat to villous mucinous epithelium without marked cytologic atypia or increased mitotic activity, and intraluminal mucin was present. The final diagnosis was LAMN measuring 10 cm in length and 2.2 cm in diameter, with an uninvolved appendiceal base and no extra-appendiceal mucin, microscopic perforation, invasive adenocarcinoma, or high-grade malignant features. The uninvolved appendiceal base and absence of extra-appendiceal mucin or microscopic perforation support a favorable pathological risk profile; however, margin involvement in LAMN remains clinically relevant when present and may influence decisions regarding additional surgery or surveillance [16].

Postoperative surveillance should be individualized according to pathological risk factors, including perforation, extra-appendiceal mucin, cellular mucin, positive margins, peritoneal involvement, and tumor markers when available. In addition, colonoscopic evaluation should be considered during follow-up to assess for synchronous colorectal lesions, particularly in patients with appendiceal mucinous neoplasms. Active surveillance has been proposed for selected low-risk patients after resection of LAMN, particularly when there is no peritoneal dissemination and complete resection has been achieved [17]. Structured surveillance programs have also been used to monitor progression to pseudomyxoma peritonei, supporting the concept that follow-up should be risk-adapted rather than uniform for all patients [18]. This case reinforces the importance of considering appendiceal mucocele and underlying LAMN in patients with chronic right lower quadrant pain and cystic appendiceal lesions. It also highlights the value of computed tomography for operative planning, meticulous intraoperative handling to prevent rupture, and risk-adapted follow-up, because surveillance strategies for LAMN and pseudomyxoma peritonei continue to evolve [19]. This report is limited by the short postoperative follow-up period and by the lack of regional lymph node assessment in the available pathological report. Therefore, long-term oncological outcomes and surveillance findings are not yet available.

## Conclusions

LAMN may present with nonspecific and chronic symptoms, making preoperative diagnosis challenging. In this case, persistent right lower quadrant pain, normal laboratory findings, and imaging evidence of a dilated appendix with low-attenuation intraluminal content and mural calcification supported the preoperative diagnosis of appendiceal mucocele. Careful surgical planning and intraoperative handling were essential to achieve complete resection while avoiding appendiceal rupture and mucin spillage. Right hemicolectomy was performed because limited appendectomy was considered unsafe due to the close association of the lesion with the cecal region. Final histopathological examination confirmed LAMN measuring 10 cm in length and 2.2 cm in diameter, with an uninvolved appendiceal base and no extra-appendiceal mucin, microscopic perforation, invasive adenocarcinoma, or high-grade features. This case emphasizes the importance of individualized surgical decision-making and risk-adapted postoperative follow-up in patients with suspected appendiceal mucinous lesions, particularly when final histopathology confirms LAMN and long-term oncologic outcomes are not yet available.
